# Oxygen Ion Concentration Distribution Effect on Bipolar Switching Properties of Neodymium Oxide Film’s Resistance and Random Access Memory Devices

**DOI:** 10.3390/nano15060448

**Published:** 2025-03-15

**Authors:** Kai-Huang Chen, Ming-Cheng Kao, Hsin-Chin Chen, Yao-Chin Wang

**Affiliations:** 1 Department of Electronic Engineering, Cheng Shiu University, Kaohsiung 83347, Taiwan; 0662@gcloud.csu.edu.tw (H.-C.C.); 0644@gcloud.csu.edu.tw (Y.-C.W.); 2 Graduate Institute of Aeronautics, Department of Information and Communication Engineering, Chaoyang University of Technology, Taichung 413310, Taiwan

**Keywords:** RRAM, neodymium oxide, oxygen ion concentration distribution effect, films, sputtering

## Abstract

In this study, the bipolar resistance switching behavior and electrical conduction transport properties of a neodymium oxide film’s resistive random access memory (RRAM) devices for using different top electrode materials were observed and discussed. Different related electrical properties and transport mechanisms are important factors in applications in a film’s RRAM devices. For aluminum top electrode materials, the electrical conduction mechanism of the neodymium oxide film’s RRAM devices all exhibited hopping conduction behavior, with 1 mA and 10 mA compliance currents in the set state for low/high voltages applied. For TiN and ITO (Indium tin oxide) top electrode materials, the conduction mechanisms all exhibited ohmic conduction for the low voltage applied, and all exhibited hopping conduction behavior for the high voltage applied. In addition, the electrical field strength simulation resulted in an increase in the reset voltage, indicating that oxygen ions have diffused into the vicinity of the ITO electrode during the set operation. This was particularly the case in the three physical models proposed, and based on the relationship between different ITO electrode thicknesses and the oxygen ion concentration distribution effect of the neodymium oxide film’s RRAM devices, they were investigated and discussed. To prove the oxygen concentration distribution expands over the area of the ITO electrode, the simulation software was used to analyze and simulate the distribution of the electric field for the Poisson equation. Finally, the neodymium oxide film’s RRAM devices for using different top electrode materials all exhibited high memory window properties, bipolar resistance switching characteristics, and non-volatile properties for incorporation into next-generation non-volatile memory device applications in this study.

## 1. Introduction

Memory types were broadly divided into volatile and non-volatile memory. Volatile memory refers to memory in which stored data are erased when the external power supply is removed. The most common examples are SRAM and DRAM. SRAM has a fast read speed and low power consumption. In contrast to the six transistors required for each bit of data in SRAM, DRAM can process each bit using only one capacitor and one transistor. This implies that DRAM has greater capacity and density per unit volume, which results in a low manufacturing cost [1,2,3,4,5,6,7,8,9,10,11,12,13,14,15,16].

Non-volatile memory is commonly used to store information because the stored data are not erased when the external power supply is removed. Traditional non-volatile memory includes flash memory and ROM. However, traditional non-volatile memory exhibits the disadvantages of a slow read/write speed. In recent years, new types of non-volatile memory have been developed that combine the advantages of volatile memory with non-volatile characteristics to improve their disadvantages. These memories feature fast read and write speeds, low power consumption, and better read-endurance properties. To date, the following four types of emerging volatile memories have been developed: magnetoresistive random access memory (MRAM), phase change random access memory (PCRAM), ferroelectric random access memory (FeRAM), and resistive random access memory (RRAM) [17,18,19,20,21,22].

RRAM devices are currently the focus of much research attention because their structures use a metal/insulator/metal (MIM) sandwich structure. The RRAM device’s structure overlapped and reduced the finished area and memory capacity. Compared with other emerging memories, RRAM devices have the advantages of low write voltage, fast read speed, and high integration density. Another study indicates that the Al/Nd_2_O_3_/ITO structure also facilitates the operation of the Nd_2_O_3_ film’s resistive memory at a high frequency and improves the switching ratio [23]. In addition to the study of the dielectric layer, the contact between the metal and the dielectric layer in the MIM structure of the RRAM was also a key point of investigation as it directly affects the characteristics of the RRAM. Currently, there is a significant body of research focusing on the effect of different electrode materials on the electrical characteristics of RRAM devices.

To discuss the oxygen concentration distribution effect in oxygen-rich ITO electrodes, the low set/reset voltages of the neodymium oxide film’s RRAM devices for different ITO electrode thicknesses were discussed and investigated. Additionally, the extremely different set voltage might be discussed based on the electric field concentration effect at the tip of the RRAM resistance wire and the oxygen ion concentration distribution effect. Finally, we varied the different-thickness ITO electrodes to illustrate the influence of different electrode materials on bipolar switching characteristics for the neodymium oxide film’s resistance of its random access memory devices on the set voltage mechanism and the electrical field strength simulation method [23,24,25,26].

## 2. Experimental Section

In this study, the neodymium oxide (Nd_2_O_3_) films of the metal–insulator–metal (MIM) resistance random access memory devices were synthesized using the radio frequency (rf) sputtering method. Furthermore, the neodymium oxide target was positioned at a distance of approximately 5 cm from the substrate. In order to eliminate the defects inherent to the oxide target and ensure the stability of the plasma during the deposition process, a pre-sputtering time of 20 min was maintained under an argon atmosphere for the as-deposited films. The radio frequency (rf) sputtering power applied to the 3-inch neodymium oxide targets was 160 watts of the Ar + 25% O_2_ ratio. Following the pre-deposition process at room temperature, the as-deposited neodymium oxide films were subjected to rapid temperature annealing (RTA) processing. Subsequently, the as-deposited neodymium oxide films underwent a post-treatment process, which involved different rapid thermal annealing processing annealing temperatures and 30 s of treatment. In addition, much information on the neodymium oxide (Nd_2_O_3_) film’s materials used to create the tested devices was prepared and treated, and some analysis techniques were presented from a previous study [23].

The metal–insulator–metal (MIM) structure sample devices, which were prepared using different top electrode materials, were divided for the purpose of discussing the potential for bipolar switching properties of the Al/neodymium oxide/ITO, TiN/neodymium oxide/ITO, and ITO/neodymium oxide/ITO RRAM devices. These devices were prepared by means of dc and rf sputtering technology, and their structures are illustrated in the subsequent figure. The dc sputtering power was set to 200 W for titanium nitride. Subsequently, the aluminum films were deposited as the top electrode using thermal evaporation technology. In addition, the ITO films were prepared using the rf sputtering technology of 100 W and the Ar + 25% O_2_ ratio gas. The top electrode diameter size of the film’s RRAM devices was about 0.05 cm.

In addition, the surface microstructure of the neodymium oxide film was examined using field emission scanning electron microscopy (FE-SEM). The images and cross-sectional structure were obtained by a transmission electron microscope (TEM), the Philips Tecnai G2 F20 (FEG-STEM). The preferred orientation of the films was determined and observed through X-ray diffraction (XRD) analysis. The *I*-*V* switching characteristics of the neodymium oxide film’s RRAM devices were determined and observed using an Agilent B1500 semiconductor parameter analyzer. The voltage sweep rate was 0.01 V/s. Additionally, the electrical properties of the precision power measurement equipment were employed to assess the *I*-*V* curves of the various compliance currents and different top electrode materials for the oxygen ion concentration distribution effect of the film’s RRAM device using the finite element method for simulation software (COMSOL multiphysics version 6.3).

## 3. Results and Discussion

**Figure 1:**
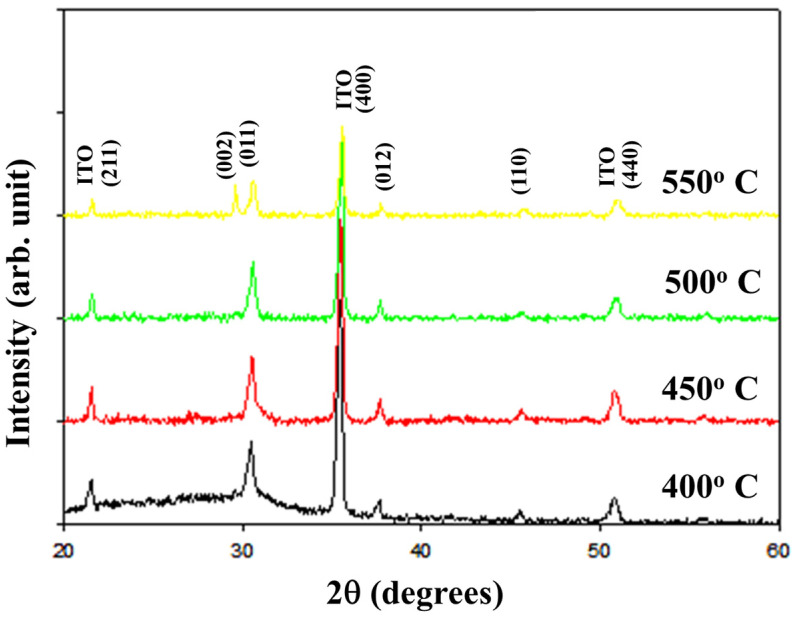
The X-ray diffraction (XRD) patterns of the neodymium oxide films were examined at different annealing temperatures, ranging from 400 °C to 550 °C.

Figure 1 depicts the X-ray diffraction (XRD) patterns of the (011), (012), and (110) preferred peaks of the as-deposited neodymium oxide films on the ITO substrate subjected to different annealing temperatures. The (211), (400), and (440) ITO peaks were also observed. It was observed that all the films exhibited a polycrystalline structure irrespective of the annealing temperature applied. The smallest full width at a half maximum (FWHM) value for the (011) preferred peaks of the neodymium oxide films at different annealing temperatures was measured. In addition, the (011) and (012) preferred peak intensities of the films were continuously slow in increasing from a 450 °C to 500 °C annealing temperature. The crystallinity of the neodymium oxide films was prepared using an annealing temperature of 450 °C and exhibited the optimal preferred (011) peak relative to the other temperature treatments. However, the (011) and (002) peaks were found and observed in 2θ = 29° at an annealing temperature of 550 °C. In addition, the ITO substrate exhibited the upper limit condition for rapid thermal annealing processing (RTP) at a process temperature of 550 °C because of its melt point and substrate deformation. Therefore, the 450 °C annealing film’s characteristics of the annealing temperature for the following RRAM device component manufacturing were chosen as the measurement and for analysis.

**Figure 2:**
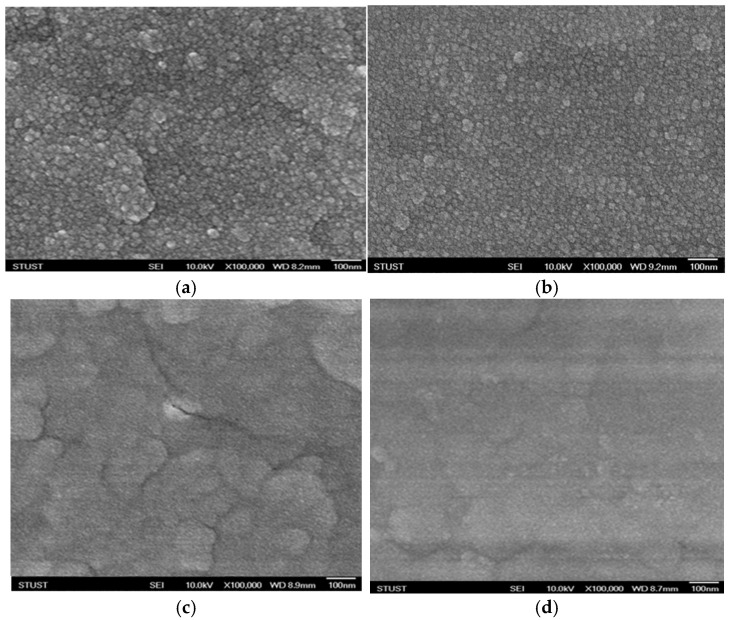
The micro-structure of the neodymium oxide films at varying annealing temperatures for (**a**) 400 °C, (**b**) 450 °C, (**c**) 500 °C, and (**d**) 550 °C.

The microstructure of the neodymium oxide films was observed using scanning electron microscopy (SEM) to examine the surface morphology at different annealing temperatures, ranging from 400 °C to 550 °C. The specimens of the films exhibited a thickness of approximately 15 nm, as seen in the TEM images, and displayed a dense and uniform grain structure. Additionally, circular plates and oval grains were observed. As illustrated in Figure 2, the microstructure and grain size of the neodymium oxide films subjected to annealing at 450 °C exhibited a predominantly round or circular morphology for other annealing temperature samples. The grain size of the neodymium oxide films was found to be approximately 30 ± 5 nm.

**Figure 3:**
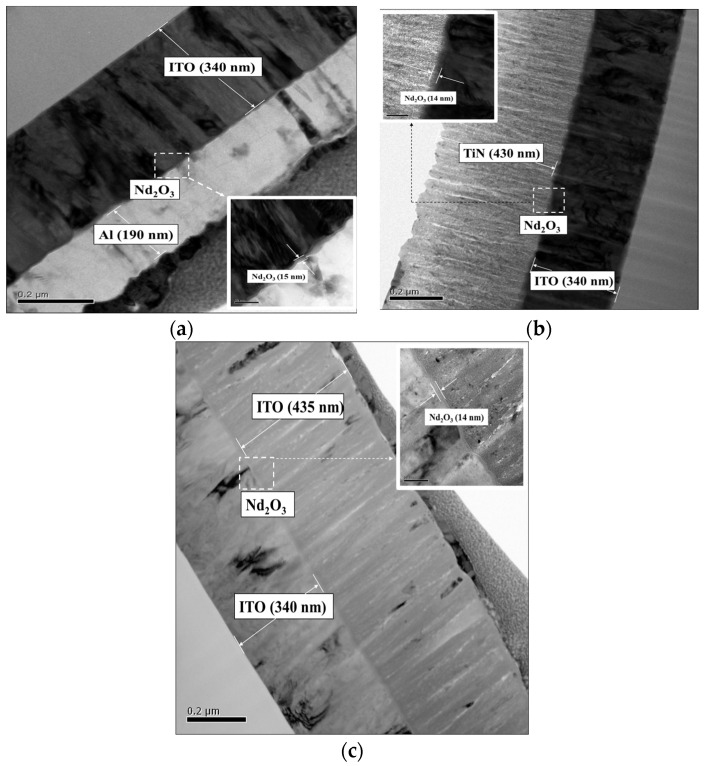
The cross-section structure of the neodymium oxide film’s RRAM devices with different top electrode materials for (**a**) Al, (**b**) TiN, and (**c**) ITO material.

Figure 3 presents the cross-section of the neodymium oxide films of the RRAM devices for the different top electrode materials (Al, TiN, and ITO). In Figure 3a, the thicknesses of the Al (top electrode), neodymium oxide films, and ITO substrate of the RRAM device’s structure were approximately 190 nm, 15 nm, and 340 nm, respectively. In Figure 3a, the thickness of the AlOx between the Al and neodymium oxide film was calculated and obtained to be 1–2 nm by using the online mapping of TEM morphology. In Figure 3b,c, the TiN and ITO top electrodes of the RRAM device’s structure exhibited thicknesses of approximately 430 nm and 340 nm, respectively. Subsequent observations and measurements of the *I*-*V* curves to determine the bipolar switching properties and electrical conduction mechanism of the neodymium oxide films revealed their significance for applications in non-volatile RRAM devices.

**Figure 4:**
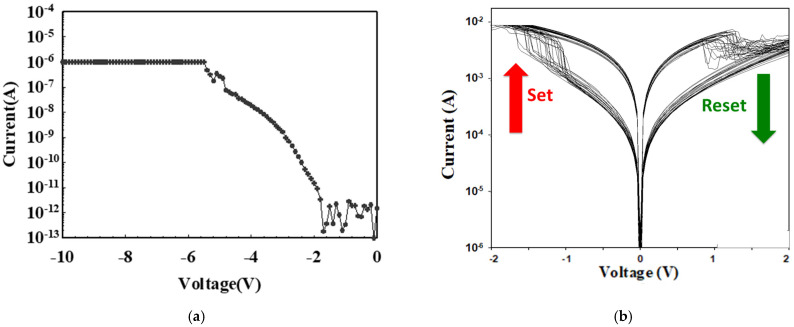
The *I*-*V* curves of the neodymium oxide film’s RRAM devices for (**a**) the initial forming process, and (**b**) the ITO/film/ITO structure from 100 measurements.

The properties of the neodymium oxide film’s RRAM devices with the initial forming process at the set and reset states are illustrated in Figure 4a. The purpose of the mechanism was to establish the resistance wire state in the film’s RRAM devices for the high voltage applied. The initial forming process was conducted at a voltage of approximately 6 V. In the set process, the neodymium oxide film’s RRAM device was transferred to a low resistance state (LRS), whereby a high negative bias was applied in excess of the set voltage during set and reset processing. In the reset process, the operation current exhibited a continuous decrease from the low resistance state (LRS) to the high resistance state (HRS) when a positive bias was applied over the reset voltage. To obtain the stable *I*-*V* operating switching condition, the set and reset processes of the neodymium oxide films of the ITO/film/ITO structure RRAM devices were repeated 100 times in Figure 4b. As illustrated in Figure 4b, the current compliance was 10 mA. The set and reset voltages of the neodymium oxide film’s RRAM device were approximately −0.5 V and −0.5 V, respectively.

**Figure 5:**
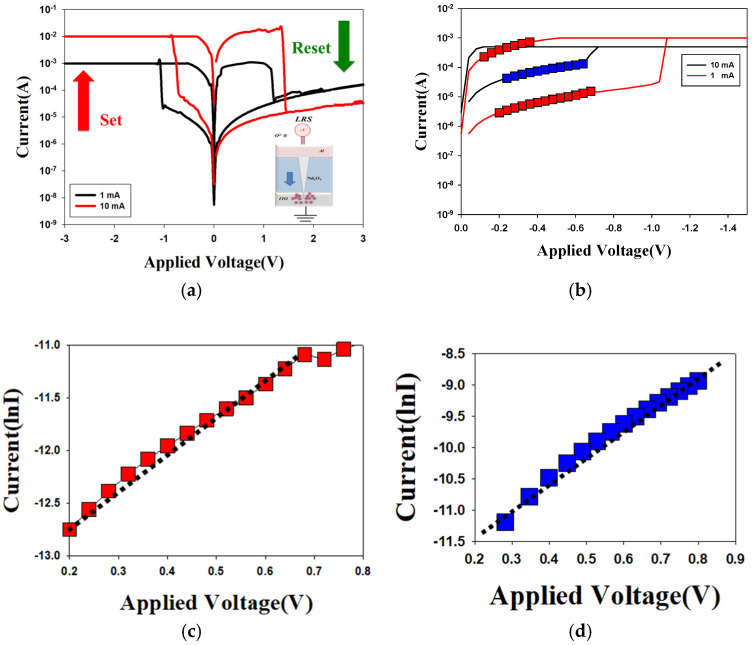
The *I*-*V* curves of the neodymium oxide film’s RRAM device with aluminum top electrode under different (**a**) compliance current processes, and (**b**) conduction mechanisms, (**c**) hopping conduction of LRS, and (**d**) hopping conduction of HRS.

**Figure 6:**
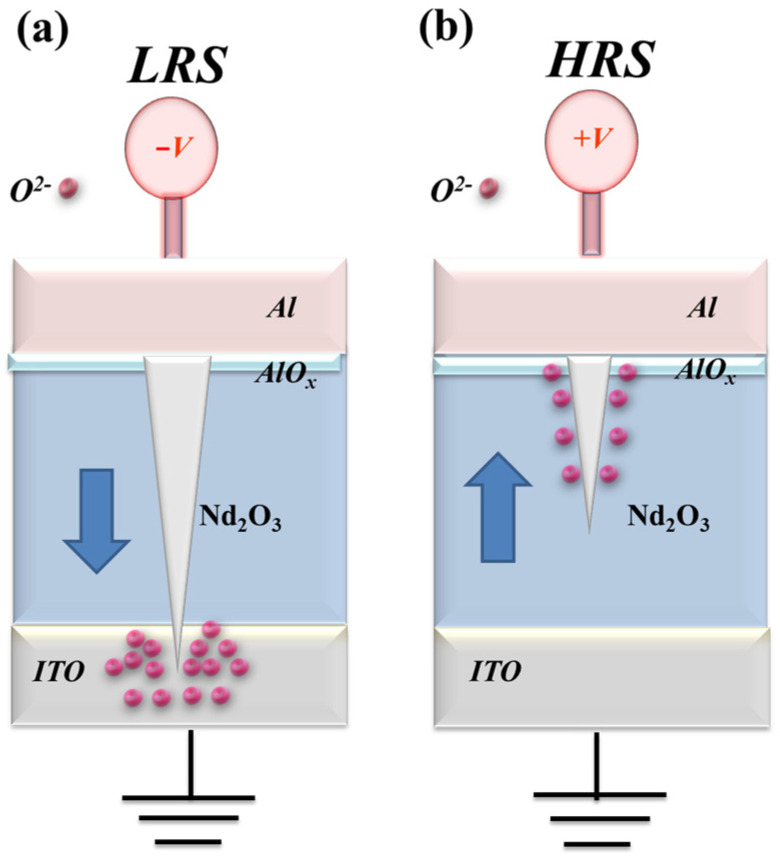
The electrical transfer mechanisms and initial metallic filament path model of the neodymium oxide film’s RRAM devices for aluminum electrode for (**a**) set, and (**b**) reset state.

Figure 5 presents the *I*-*V* curves of the neodymium oxide film’s RRAM device using an aluminum top electrode under different (a) compliance current processes and (b) conduction mechanisms. The applied compliance currents for the neodymium oxide film’s RRAM device were 1 mA and 10 mA, respectively. The set and reset voltages of the RRAM device for a compliance current of 1 mA were approximately less than −1 V and 1 V, respectively. For a compliance current of 10 mA, the memory windows exhibited a high 10^4^ ratio and a low set voltage. This result may be indicative of the low set voltage of high switch cycles induced by the high compliance current. Figure 5b depicts the *I*-*V* curves of the neodymium oxide film’s RRAM device with an aluminum top electrode for different conduction mechanisms. The neodymium oxide film’s RRAM device for 1 mA and 10 mA compliance currents both exhibited the hopping conduction mechanism in a low set voltage, as illustrated in Figure 5b. In addition, the electrical conduction mechanism of the film’s RRAM device’s 10 mA compliance current exhibited hopping conduction behavior for all LRS/HRS, as shown in Figure 5c,d. The electrical transfer mechanisms and initial metallic filament path model of the neodymium oxide film’s RRAM devices for the aluminum electrode in the set/reset state are also presented and described in Figure 6.

In Figure 6a, the oxygen vacancies exist in the interface region between the AlOx top electrode/films of the RRAM device and continue to accumulate for LRS states, as described and presented. The physical conduction model showed the continuing oxidation reaction effect of the thin metal metallic filament path in high positive voltage in Figure 6b. The electrical transfer mechanisms and initial metallic filament path model of the film’s RRAM devices for the AlOx top electrode in the set/reset state are described in Figure 6. According to previous research, the electrical conduction mechanism of a film’s RRAM devices all exhibited hopping conduction behavior for all LRS/HRS, as shown in Figure 5c,d; this was also proved in Figure 6 [27,28].

**Figure 7:**
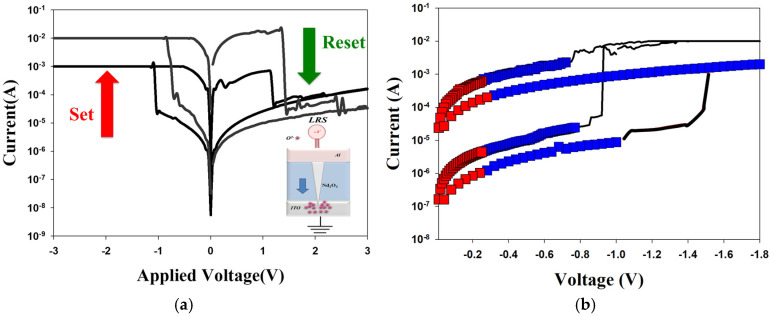

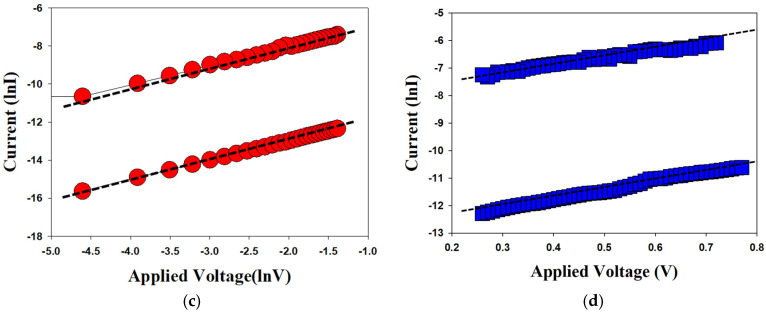
The *I*-*V* curves of the neodymium oxide film’s RRAM device with a TiN electrode under different (**a**) compliance current processes and (**b**) conduction mechanisms (**c**) ohmic conduction for low electrical voltage, and (**d**) hopping conduction for high electrical voltage.

**Figure 8:**
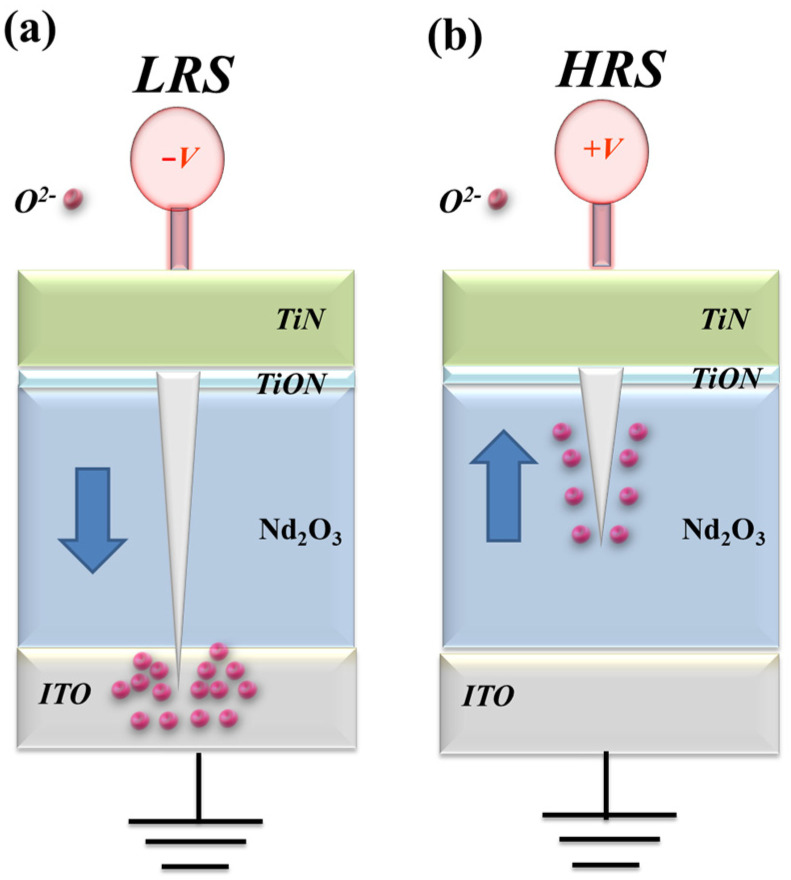
The electrical transfer mechanisms and initial metallic filament path model of the neodymium oxide film’s RRAM devices for TiN electrode for (**a**) set, and (**b**) reset state.

Figure 7 presents the *I*-*V* curves of the neodymium oxide film’s RRAM device with a TiN top electrode under different (a) compliance current processes and (b) conduction mechanisms. The set and reset voltages of the RRAM device with a TiN top electrode for a 1 mA compliance current were approximately less than −0.5 V and 0.5 V, respectively. At the 10 mA compliance current, the memory windows exhibited a high 10^3^ ratio and low set voltage. The result may also be indicative of a low set voltage resulting from high switch cycles induced by a high compliance current. Figure 7b depicts the *I*-*V* curves of the neodymium oxide film’s RRAM device with a TiN top electrode under different conduction mechanisms. The neodymium oxide film’s RRAM device with 1 mA and 10 mA compliance currents all exhibited a hopping conduction mechanism at low set voltages and an ohmic conduction mechanism at high applied voltages, as shown in Figure 7c,d. The oxygen vacancies existed in the interface region between the TiN bottom/film of the RRAM device and gradually accumulated for LRS states. The physical conduction model provided a continuing oxidation reaction of the thin metal metallic filament path in a high positive voltage. Thin metal metallic filaments were affected by oxygen atoms near the bottom electrode area. The electrical transfer mechanisms and initial metallic filament path model of the film’s RRAM devices for the TiN electrode in the set/reset state are described in Figure 8 [27,28]. In the TiN top electrode RRAM devices compared to an aluminum top electrode, the electrical conduction mechanism exhibited a hopping conduction mechanism in low voltage applied, which might be caused by different barriers in the top metal electrode’s and neodymium oxide film’s interface of the RRAM device structure [29].

**Figure 9:**
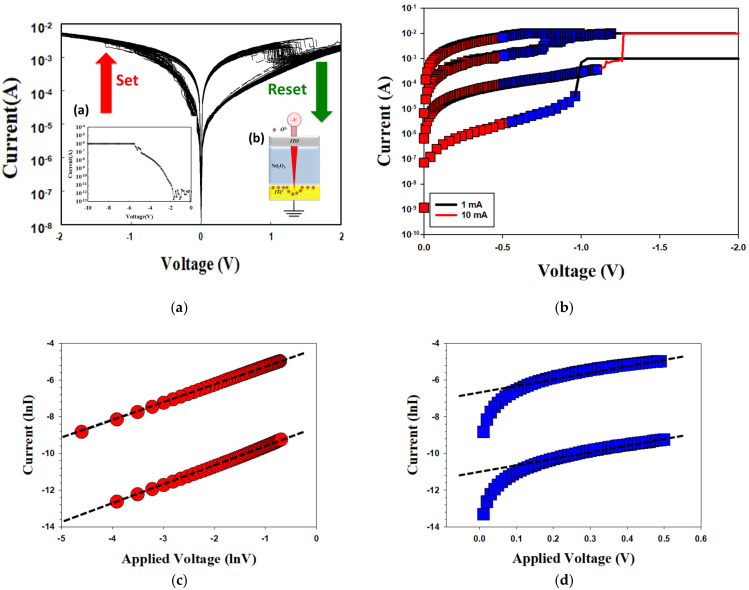
The *I*-*V* curves of the neodymium oxide film’s RRAM device with an ITO electrode under different (**a**) compliance current processes and (**b**) conduction mechanisms (**c**) ohmic conduction, and (**d**) hopping conduction.

**Figure 10:**
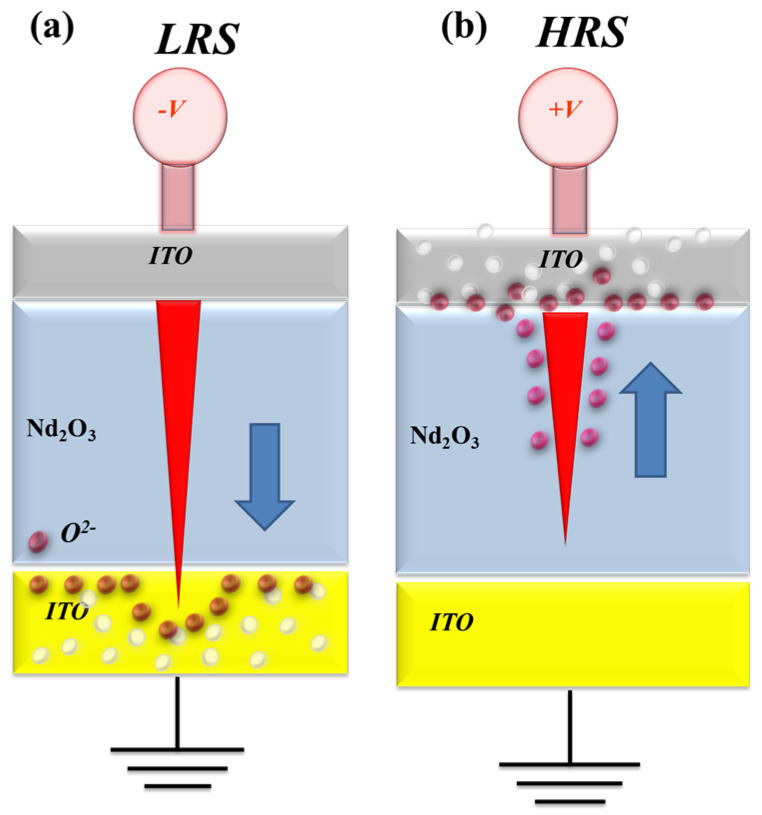
The electrical transfer mechanisms and initial metallic filament path model of the neodymium oxide film’s RRAM devices for ITO electrode for (**a**) set, and (**b**) reset state.

Figure 9 also presents the *I*-*V* curves of the neodymium oxide film’s RRAM device with a 300 nm ITO top electrode under different (a) compliance current processes and (b) conduction mechanisms. The set and reset voltages of the RRAM device with an ITO top electrode and 1 mA compliance current were approximately less than −0.2 V and 0.5 V, respectively. At a compliance current of 1 mA, the memory windows demonstrated a high 10^3^ ratio and a low set voltage. However, the memory windows exhibited a low 10^2^ ratio, with a 10 mA compliance current. This result might also indicate the involvement of oxygen ions from the ITO electrode in the electron conduction path of the films. Figure 9b depicts the *I*-*V* curves of the neodymium oxide film’s RRAM device with an ITO top electrode under different conduction mechanisms. The neodymium oxide film’s RRAM device exhibited a hopping conduction mechanism at low set voltages and an ohmic conduction mechanism at high applied voltages for 1 mA and 10 mA compliance currents, as shown in Figure 9c and d, respectively. The physical conduction model also provided an elliptical pattern in the depletion region, which was formed by oxygen ions and vacancies that gradually accumulated in the ITO top electrode of the film’s RRAM device for LRS. Additionally, the conductive metal continuously and sharply adsorbed oxygen atoms and vacancies in the ITO electrode in HRS. In Figure 10, the electrical transfer mechanisms and initial metallic filament path model of the film’s RRAM devices for the ITO electrode are also described [27,28].

**Figure 11:**
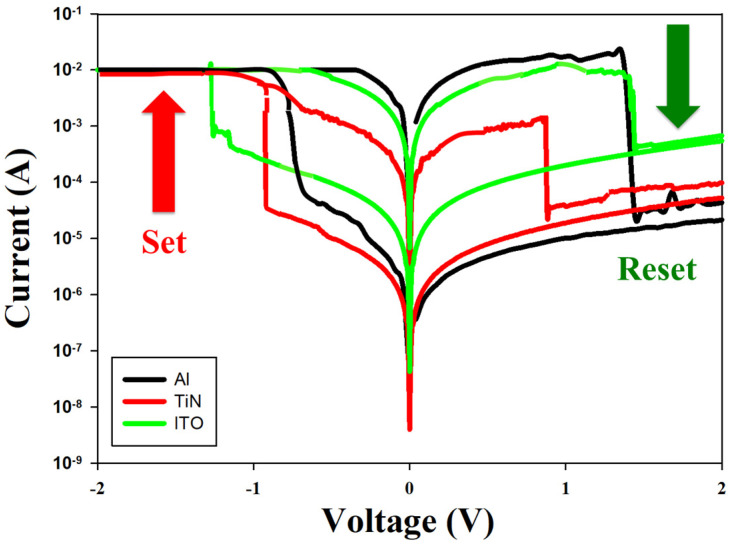
The *I*-*V* curves of the neodymium oxide film’s RRAM device for different top electrode materials (Al, TiN, and ITO).

The *I*-*V* curves of the neodymium oxide film’s RRAM device with different top electrode materials for aluminum, TiN, and ITO materials are presented in Figure 11. For the compliance current of 10 mA, the *I*-*V* curves of the neodymium oxide film’s RRAM device for the ITO electrode exhibited a symmetry memory window within the set/reset voltage range. The observed symmetry of the *I*-*V* curves might be attributed to the barrier height at the interface between the films and the ITO electrode in the RRAM device, which employs the same top and bottom ITO electrode materials [23]. In contrast, the top electrode of the RRAM device using aluminum or TiN exhibited asymmetric *I*-*V* curves. The asymmetry of the *I*-*V* curves in the set/reset voltage ranges might be attributed to the disparate barrier heights of the films and the ITO electrode interface. The memory window and set/reset voltage were measured and defined based on an on-conduction current of 10^−3^ A. As shown in Figure 11, in a set state, the memory window ratios of the neodymium oxide film’s RRAM devices for a read voltage of 0.5 V were approximately 10^3^, 10^2^, and 10^2^ for aluminum, TiN, and ITO top materials, respectively.

**Figure 12:**
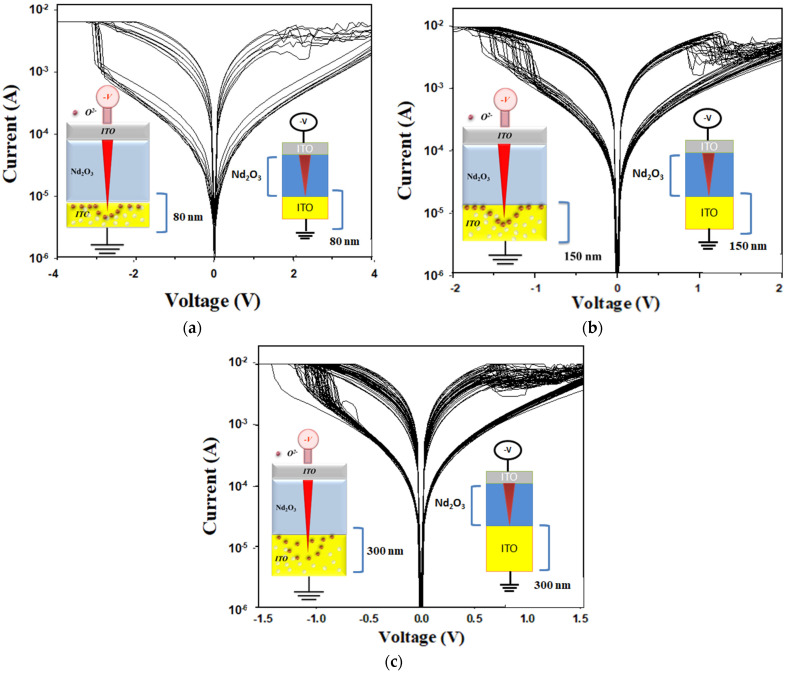
The *I*-*V* curves of the neodymium oxide film’s RRAM device using ITO as the top electrode materials with thicknesses of (**a**) 80 nm, (**b**) 150 nm, and (**c**) 300 nm.

To compare the effects of oxygen ion concentration distribution on the electrical properties of the film’s RRAM devices, different ITO top electrode material thicknesses of 80, 150, and 300 nm were fabricated and demonstrated in Figure 12. In the insert of Figure 12a–c, the left image depicts the electron transfer model, while the right image represents the MIM structure of the neodymium oxide film’s RRAM device. In Figure 12a, the set and reset voltages of the RRAM devices for the 80 nm ITO top electrode materials were observed to be −1.5 V and 1.5 V, respectively. In Figure 12b,c, the set and reset voltages decreased as the top ITO electrode thickness increased from 80 nm to 300 nm. In addition, the real physical model and the RRAM device’s structure for the set state are also presented in the insert of Figure 12a. In addition, the set and reset voltages of the RRAM devices decreased as the ITO top electrode material’s thickness increased and might have contributed to the oxygen ion concentration distribution effect from 80 nm to 300 nm, as shown in Figure 12b,c.

**Figure 13:**
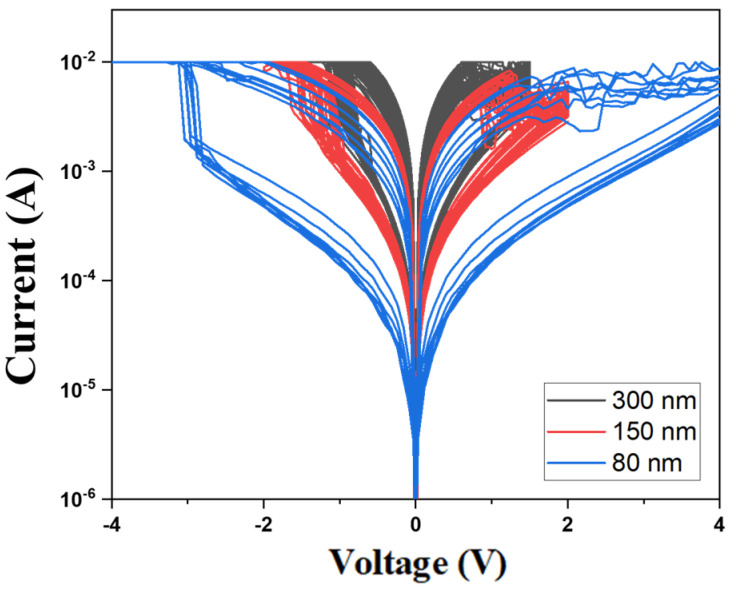
The *I*-*V* curves of the neodymium oxide film’s RRAM device using ITO as the top electrode materials with different thicknesses.

Figure 13 presents the *I*-*V* curves of the neodymium oxide film’s RRAM device using ITO as the top electrode material, with different thicknesses from 100 cycle time measurements. The neodymium oxide film’s RRAM device, with a thickness of 80 nm, exhibited the maximum memory window in the order of 10^2^ in both set and reset states. However, the neodymium oxide film’s RRAM device, with a thickness between 80 and 150 nm, exhibited a relatively narrow memory window in the order of 10^2^. As shown in Figure 13, the set and reset voltages of the RRAM devices decreased from −1.5 V to 0.5 V as the thickness increased from 80 nm to 300 nm. To discuss the set/reset voltage versus the oxygen ion’s concentration distribution effect on the bipolar switching properties for different ITO top electrode materials thicknesses of RRAM devices, we used a finite element method simulation software.

**Figure 14:**
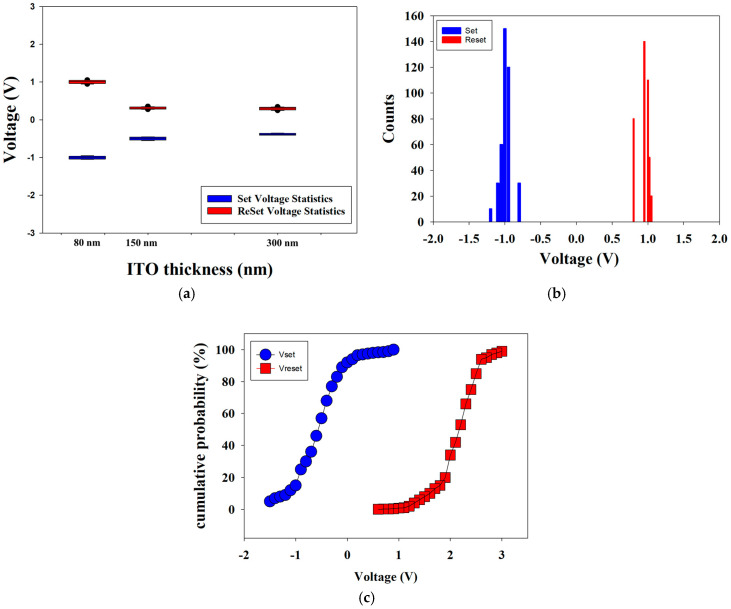
The set/reset voltage statistics of the neodymium oxide film’s RRAM device using ITO as the top electrode materials for 80 nm with (**a**) different thicknesses compared, (**b**) counts distribution, and (**c**) cumulative probability.

Figure 14 shows the set and reset voltages of the neodymium oxide film’s RRAM devices using ITO as the top electrode material with different thicknesses. Figure 14b,c presents the statistical results of the distribution of the set/reset voltage measured in RRAM devices using an 80 nm ITO electrode thickness that was measured 400 times. In Figure 14b, the set and reset voltage values are shown for the range of applied voltages from −1 to 1 V. In addition, the statistical results of the cumulative probabilities for the resistive switching properties of the RRAM devices are also observed in Figure 14c. As shown by the set statistical results in Figure 14c, an increase in thickness correlates with a reduction in the set voltage. This suggests that oxygen ions are driven into the ITO film’s electrode, resulting in the creation of additional oxygen vacancies during the set process. As the thickness of the ITO continues to increase, the oxygen concentration distribution expands over a larger area of the ITO electrode. This may be a contributing factor to the low set voltage operation of the neodymium oxide film’s RRAM in the set process. The obvious symmetry of the *I*-*V* curves of the RRAM device for the different top electrode material thicknesses was also caused and proved by the set/reset voltage properties in the set/reset process [27,28,29].

**Figure 15:**
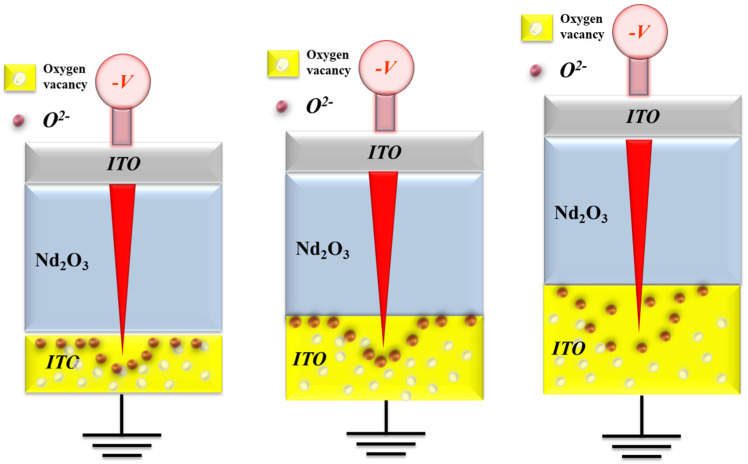
The electrical transfer mechanisms and initial metallic filament path model of the neodymium oxide film’s RRAM devices in the reset state.

In addition, the reset statistical results indicate that the reset voltage increased as the thickness of the neodymium oxide films increased. The increasing reset voltage may be due to the diffusion of oxygen ions in the range of the ITO electrode in the set operation process, as explained in Figure 15. When the high reverse bias was applied in the reset process, the oxygen ions must have been driven back to the neodymium oxide film’s switching layer. The physical model depicting the effect of oxygen concentration distribution effect in relation to different ITO electrode thicknesses is shown in Figure 15.

**Figure 16:**
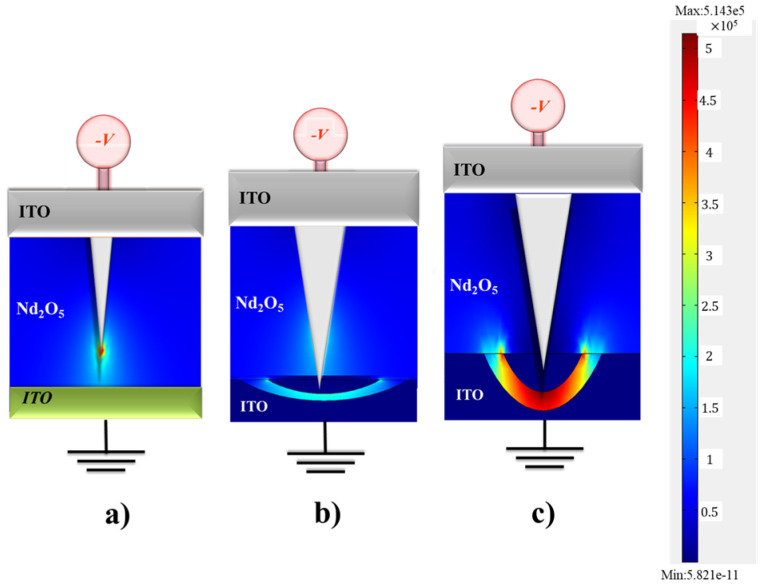
The electric field simulation diagram of the resistance wire in the neodymium oxide film’s RRAM devices for the set state of (**a**) 80 nm, (**b**) 150 nm, and (**c**) 300 nm thickness ITO electrode.

To prove that the oxygen concentration distribution expands over a larger area of the ITO electrode, finite element method comsol simulation software (version 6.3) was used to analyze the distribution of the electric field, and the Poisson equation was used for a simulation [27]. The electric field simulation diagram of the resistance wire in the neodymium oxide film’s RRAM devices for the set state of (a) 80 nm, (b) 150 nm, and (c) 300 nm thicknesses for the ITO electrode was established, as shown in Figure 16. The process of component forming established the metal filament of the conduction path, as shown in the schematic diagram in Figure 16a. In the tip of the resistance wire, the oxygen ion’s concentrated distribution of the electric field was clearly seen, and the oxygen ions were repelled into the ITO electrode, forming an elliptical depletion area with high resistance, as shown in Figure 16b’s electric field simulation diagram. In Figure 16c, the electric field simulation diagram of the resistance wire in the neodymium oxide film’s RRAM devices for the set state of 300 nm thickness for the ITO electrode clearly exhibits the oxygen ion’s concentrated distribution effect over a larger area of the ITO electrode. However, the energy required during the reset process was much greater than that required for the set process in the electric field simulation diagram.

**Figure 17:**
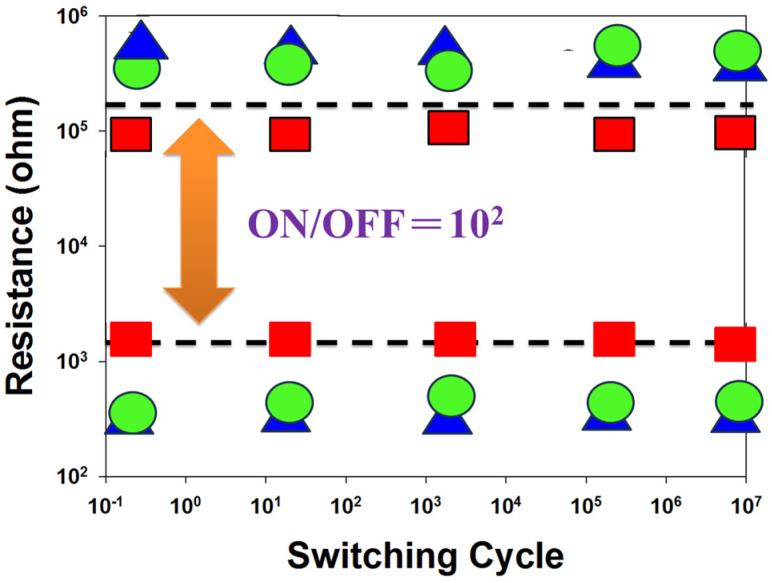
The resistance value versus time curves of the neodymium oxide film’s RRAM devices (red: ITO, green: TiN, blue: aluminum).

Figure 17 shows the switching cycling versus resistance value curves, determined by the retention and endurance measurement properties. The retention properties of the neodymium oxide film’s RRAM devices with different top electrode materials (Al, TiN, ITO) were measured to investigate their reliability for applications in non-volatile memory RRAM devices. There were no significant changes in the ON/OFF ratio switching resistance cycling versus the testing time curves in the neodymium oxide film’s RRAM devices for more than 10^2^ s in the extrapolation calculation anticipation measured.

**Figure 18:**
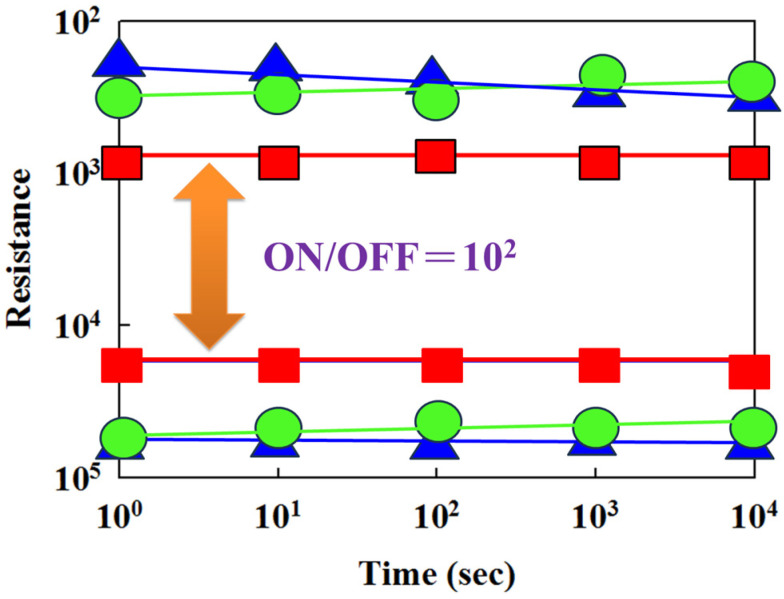
The resistance value versus switching cycle curves of the neodymium oxide film’s RRAM devices (red color: ITO, green color: TiN, blue color: aluminum).

Figure 18 presents the resistance value versus the switching cycle curves of the neodymium oxide film’s RRAM devices with ITO (red), TiN (green), and aluminum (blue) as the top electrode materials. The neodymium oxide film’s RRAM device showed no significant changes in the ON/OFF ratio switching behavior cycling versus the time curves for more than 10^2^ s in the extrapolation calculation anticipation.

To compare the difference in the film’s RRAM device for using the different top electrode materials, the electrical properties of Ea (activation energy) and the compliance current properties were calculated and obtained in Table 1. To explore the electrical conduction behavior of the initial metallic filament forming conduction, the ohmic conduction and hopping conduction mechanisms were determined by *I*-*V* and ln*I–V*^1/2^ curve fitting. To calculate the *I*-*V* curve, the ohmic conduction mechanism equation was transformed from the *I*-*V* curves fitting the RRAM devices. For the ohmic conduction,

(1) $$J=E\exp\left(\frac{-\Delta E_{ac}}{kT}\right)$$

where *Eac* is the electron activation energy, *E* is electrical field, k is the Boltzmann constant, and T is the absolute temperature.

In addition, to calculate the $\ln\left(\frac{I}{T^{2}}\right)$−$\sqrt{V}$ curve, the hopping conduction mechanism equation was transformed to the *I*-*V* curves fitting the RRAM devices. For the hopping conduction,

(2) $$J=\alpha nvq\;\times \;\exp\left(\frac{-U}{kT}+\frac{q\alpha E}{kT}\right)$$

where *E* is electrical field, and *J* is current density, *U* is barrier height of hopping, *v* is intrinsic vibration frequency, *n* is density of space charge, and α is mean hopping distance, respectively [23].

Table 1 presents the structure and compliance current of the different film’s RRAM devices. In this study, the electron activation energy in the case of the electrical conduction mechanism was extracted from the *I*-*V* curves and compared with the values observed in the literature summarized in Table 1. The activation energy values of the film’s RRAM devices were about 128 meV and 94 meV for using the 1 mA and 10 mA compliance current, respectively. From Table 1, the low activation energy values of the film’s RRAM devices were induced by the high compliance current.

**Table 1:** The activation energy (Ea) for compared on the structure and the compliance current of the various film’s RRAM devices.

.ROW	Structure	Ea (Activation Energy)	Compliance Current	REF
1	Pt/Zn:SiO_2_/TiN	153.3 meV	10 μA	[30]
2	Pt/Zn:SiO_2_/TiN	68.3 meV	100 μA	[30]
3	Pt/Sn:SiO_2_/TiN	122 meV	10 μA	[31]
4	Pt/Sn:SiO_2_/TiN	58.8 meV	100 μA	[31]
5	Al/BST/ITO	138 meV	5 mA	[19]
6	Al/BST/ITO	117 meV	10 mA	[19]
7	Al/ITO_X_:SiO_2_/TiN	121 meV	10 mA	[28]
8	ITO/Nd_2_O_3_/ITO	128 meV	1 mA	This work
9	ITO/Nd_2_O_3_/ITO	94 meV	10 mA	This work

Table 2 presents the set/reset voltage and memory windows versus different compliance current properties on the structure of the various film’s RRAM devices. Compared to previous studies, the low set/reset voltage might be caused by the switching thin film’s oxide layer thickness (15 nm) in this study. The low set/reset voltages of the ITO/Nd_2_O_3_/ITO (top electrode 300 nm thickness) RRAM devices were calculated to be −0.2 V/0.5 V, respectively. In Table 2, for the aluminum and TiN top electrodes, we summarized and found that the set/reset voltage of the film’s RRAM devices might also be fabricated and modified by using different top ITO electrode material thicknesses.

## 4. Conclusions

In conclusion, the bipolar switching behavior and electrical conduction transport properties of the neodymium oxide film’s resistive random access memory (RRAM) devices with different top electrodes were observed and discussed. The conduction mechanisms in *I*-*V* curves of the neodymium oxide film’s RRAM device with an aluminum top electrode under different compliance current processes all exhibited a hopping conduction in set states. On the contrary, the conduction mechanisms of the RRAM device with TiN and ITO top electrodes under different compliance current processes all exhibited ohmic conduction for a low electrical voltage and hopping conduction for a high electrical voltage.

Additionally, the two main factors underlying the extremely small set voltage, the electric field that was affected at the tip of the RRAM resistance wire and the oxygen ion concentration distribution effect, were also observed. The different thicknesses of the ITO electrode illustrate the effect of the oxygen concentration distribution effect on the set voltage effect. For aluminum, TiN, and ITO top electrode materials, the memory window ratios of the neodymium oxide film’s RRAM devices were exhibited to be approximately 10^2^, 10^3^, and 10^3^, respectively. The neodymium oxide film’s RRAM device for the ITO, Al, and TiN electrode materials showed no significant changes in the ON/OFF ratio retention and endurance measurement properties for more than 10^2^ s in the extrapolation calculation anticipation. Finally, the suitable set/reset voltage, LRS/HRS resistance value, and memory windows properties of the film’s RRAM devices were modulated and prepared using different ITO top electrode thicknesses. In addition, the oxygen ion’s concentrated distribution of the electric field was clearly proved in the tip of the resistance wire, and the oxygen ions were repelled into the ITO electrode, forming an elliptical depletion area with high resistance behavior, as shown in the electric field simulation diagram.

## Figures and Tables

**Table 2:** The set/reset voltage and memory windows versus different compliance current properties for compared on the structure of the various film’s RRAM devices.

.ROW	Structure	Ser/Reset Voltage	Memory Windows	Compliance Current	REF
1	Al/Nd_2_O_3_/ITO	−1 V/1 V	10^3^	10 mA	This work
2	TiN/Nd_2_O_3_/ITO	−0.5 V/0.5 V	10^2^	10 mA	This work
3	ITO/Nd_2_O_3_/ITO (300 nm)	−0.2 V/0.5 V	10^2^	10 mA	This work
4	Al/Nd_2_O_3_/ITO	−0.5 V/0.5 V	10^2^	1 mA	This work
5	TiN/Nd_2_O_3_/ITO	−0.2 V/0.2 V	10^2^	1 mA	This work
6	ITO/Nd_2_O_3_/ITO	−0.1 V/0.3 V	10^1^	1 mA	This work
7	Pt/Zn:SiO_2_/TiN	−0.5 V/0.3 V	10^2^	10 mA	[30]
8	Pt/Sn:SiO_2_/TiN	−0.3 V/0.2 V	10^1^	10 mA	[31]
9	Al/BST/ITO	−1 V/0.5 V	10^2^	10 mA	[19]
10	Al/ITO_X_:SiO_2_/TiN	−0.5 V/0.5 V	10^2^	10 mA	[28]

## Data Availability

Data are contained within the article.
